# Effect of empagliflozin on reducing the no-reflow phenomenon in patients with ST-elevation myocardial infarction: rationale and design of the EMPA-PCI trial

**DOI:** 10.1093/ehjopen/oeaf128

**Published:** 2025-10-05

**Authors:** Fabio Solis-Jimenez, Diego Araiza-Garaygordobil, Jessy Steve Masso-Bueso, Alejandro Villalobos-Ordaz, Fernando Arellano-Juvera, Federico Arredondo-Aragon, Gabriela Melendez-Ramirez, Rafael Valdez-Ortiz, Sergio Martin Alday-Ramirez, Hugo Gerardo Rodriguez-Zanella, Luis Manuel Amezcua Guerra, Maria Alexandra Arias-Mendoza, Marco Antonio Martinez-Rios, Eduardo Agustin Arias-Sánchez, Guering Eid-Lidt

**Affiliations:** Department of Interventional Cardiology, National Institute of Cardiology Ignacio Chávez, Juan Badiano 1, Belisario Dominguez Secc 16, Tlalpan, 14080 Mexico City, Mexico; Medical School, National Autonomous University of México, Ciudad Universitaria, Coyoacán, 04510 Mexico City, Mexico; Department Coronary Care Unit, National Institute of Cardiology Ignacio Chávez, Juan Badiano 1, Belisario Dominguez Secc 16, Tlalpan, 14080 Mexico City, Mexico; Department of Interventional Cardiology, National Institute of Cardiology Ignacio Chávez, Juan Badiano 1, Belisario Dominguez Secc 16, Tlalpan, 14080 Mexico City, Mexico; Medical School, Benemerita Autonomous University of Puebla, Sur 104 Centro Histórico C.P. 72000, Puebla, Puebla, Mexico; Medical School, Autonomous University of the State of Morelos, Av. Universidad No. 1001, Chamilpa, 62210 Cuernavaca, Morelos, Mexico; Medical School, National Autonomous University of México, Ciudad Universitaria, Coyoacán, 04510 Mexico City, Mexico; Department of Magnetic Resonance Imaging, National Institute of Cardiology Ignacio Chávez, Juan Badiano 1, Belisario Dominguez Secc 16, Tlalpan, 14080 Mexico City, Mexico; Department of Nephrology, General Hospital of Mexico Eduardo Liceaga, Dr. Balmis 148, Doctores, Cuauhtémoc, 06720 Mexico City, Mexico; Department of Echocardiography, National Institute of Cardiology Ignacio Chávez, Juan Badiano 1, Belisario Dominguez Secc 16, Tlalpan, 14080 Mexico City, Mexico; Department of Echocardiography, National Institute of Cardiology Ignacio Chávez, Juan Badiano 1, Belisario Dominguez Secc 16, Tlalpan, 14080 Mexico City, Mexico; Department of Immunology, National Institute of Cardiology Ignacio Chávez, Juan Badiano 1, Belisario Dominguez Secc 16, Tlalpan, 14080 Mexico City, Mexico; Department Coronary Care Unit, National Institute of Cardiology Ignacio Chávez, Juan Badiano 1, Belisario Dominguez Secc 16, Tlalpan, 14080 Mexico City, Mexico; Department of Interventional Cardiology, National Institute of Cardiology Ignacio Chávez, Juan Badiano 1, Belisario Dominguez Secc 16, Tlalpan, 14080 Mexico City, Mexico; Department of Interventional Cardiology, National Institute of Cardiology Ignacio Chávez, Juan Badiano 1, Belisario Dominguez Secc 16, Tlalpan, 14080 Mexico City, Mexico; Medical School, National Autonomous University of México, Ciudad Universitaria, Coyoacán, 04510 Mexico City, Mexico; Department of Interventional Cardiology, National Institute of Cardiology Ignacio Chávez, Juan Badiano 1, Belisario Dominguez Secc 16, Tlalpan, 14080 Mexico City, Mexico

**Keywords:** STEMI, Empagliflozin, No-reflow phenomenon, Reperfusion Injury, PCI

## Abstract

**Introduction:**

Coronary no-reflow phenomenon occurs when cardiac tissue fails to perfuse normally despite opening of the occluded vessel. It is one of the manifestations of reperfusion injury, a series of pathological conditions associated with an increase in infarct size and adverse clinical outcomes. While there is currently no specific treatment to limit or prevent reperfusion injury, preclinical models have shown promising results with iSGLT2 inhibitors in this regard. However, there are no human studies specifically designed to evaluate the effects of empagliflozin on the no-reflow phenomenon or reperfusion injury.

**Methods and analysis:**

The EMPA-PCI is a single-centre, open-label, randomized clinical trial that compares the use of empagliflozin vs. standard treatment in reducing reperfusion injury in patients with STEMI. A total of 162 patients will be randomized to receive either 25 mg of Empagliflozin as a loading dose before angioplasty followed by 10 mg per day for three doses in the treatment group, or standard treatment in the control group. The incidence of the no-reflow phenomenon during PCI, infarct size by magnetic resonance imaging, myocardial injury biomarkers will be compared. Clinical follow-up will be conducted for 3 months following patient enrollment.

**Conclusion:**

Empagliflozin administered prior to PCI in patients with STEMI may contribute to prevent the no-reflow phenomenon and limit reperfusion injury. This could provide new insights into the cardiovascular benefits already known for SGLT2 inhibitors.

**Trial registration:**

ClinicalTrials registry. NCT06342141.

## Graphical Abstract

**Graphical Abstract:**
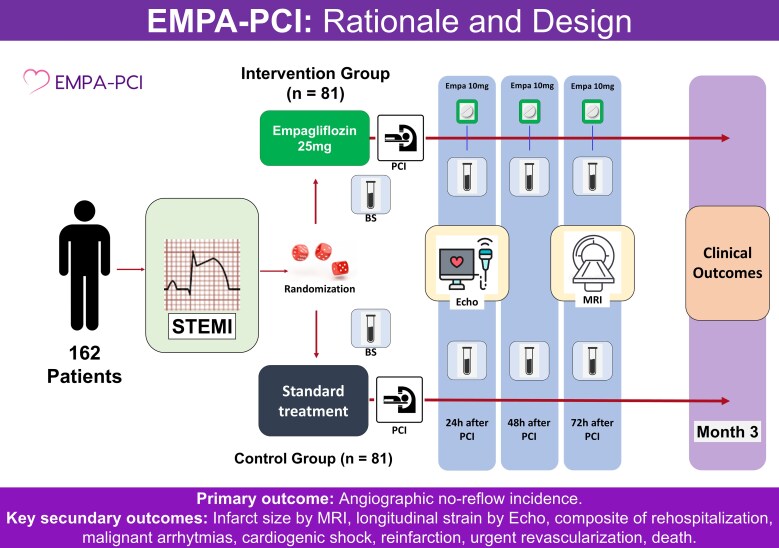

## Introduction

Acute myocardial infarction (AMI) has long been one of the leading causes of morbidity and mortality worldwide.^1^ Although reperfusion therapy in patients with ST-segment elevation myocardial infarction (STEMI) has significantly advanced, the decline in mortality observed over the past decade has markedly plateaued.^2^ A significant element underlying the adverse outcomes of AMI is reperfusion injury, a condition that has been relatively underexplored as a therapeutic target.^3^ Paradoxically, it occurs when epicardial coronary flow is restored in the affected artery, leading to increased infarct size and being associated with worse clinical outcomes.^4, 5^

One of the principal manifestations of reperfusion injury is the no-reflow phenomenon.^6^ This occurs when cardiac tissue fails to perfuse normally despite opening of the occluded vessel and this process is driven by a series of pathophysiological events involving the endothelium, inflammatory cells, and the cardiomyocytes themselves.^7, 8^ The no-reflow phenomenon may occur in up to 60% of STEMI cases and is associated with a higher risk of rehospitalization, adverse left ventricular remodeling, malignant arrhythmias, and the development of heart failure.^9, 10^ Additionally, it is an independent predictor of reinfarction and cardiovascular death.^11^

On the other hand, in the past decade, sodium-glucose co-transporter 2 (SGLT2) inhibitors, which had already demonstrated cardiovascular benefits in patients with heart failure, have also shown utility in patients with AMI by reducing the risk of heart failure-related hospitalizations.^12^ However, despite recent preclinical studies suggesting that SGLT2 inhibitors may play a significant role in reducing the no-reflow phenomenon and reperfusion injury through various mechanisms involving cardiomyocytes, the endothelium and inflammatory cells, this has not yet been confirmed in humans.^13^ To address this gap, the EMPA-PCI study was designed to evaluate the effect of empagliflozin on reperfusion injury in patients with STEMI.

## Methods and analysis

The EMPA-PCI is a randomized, parallel-group clinical trial comparing the use of empagliflozin in STEMI patients before (percutaneous coronary intervention) PCI vs. standard treatment.

### Study hypothesis and endpoints

The overall hypothesis of the EMPA-PCI trial is that administering empagliflozin before PCI will lead to better outcomes compared with standard treatment.

### Primary hypothesis

Oral administration of empagliflozin before PCI in STEMI patients will reduce the incidence of the no-reflow phenomenon compared with patients receiving standard treatment.

### Key secondary hypotheses

When compared with standard treatment, oral administration of empagliflozin before primary PCI in STEMI patients will result in:

- A reduction in infarct size (assessed by magnetic resonance imaging 72 h after PCI).
- A Higher rate of myocardial salvage index, assessed by cardiac MRI.
- Less microvascular obstruction, assessed by cardiac MRI.
- A decrease in infarct size, evaluated by the percentage of ST-segment resolution on electrocardiogram 2 h after PCI.
- A better coronary flow at the end of PCI (assessed using the TIMI flow scale).
- Better myocardial tissue-level perfusion, assessed by the Myocardial Blush Grade (MBG) at the end of the PCI procedure.
- A reduction in infarct size, evaluated by peak and area under the curve of Creatine Kinase (CK), Creatine Kinase MB (CK-MB), and troponins.
- A lower proportion of ventricular dysfunction, assessed by longitudinal strain on transthoracic echocardiography 24 h after PCI.
- Less adverse cardiac remodeling at 3-month follow-up on transthoracic echocardiography (TTE).
- A reduction in the composite outcome of rehospitalization, malignant arrhythmias, cardiogenic shock, reinfarction, urgent revascularization, and death at 3 months.

### Eligibility criteria

The inclusion and exclusion criteria are summarized in *Table 1*.

**Table 1:** Inclusion and exclusion criteria of EMPA-PCI trial

Inclusion Criteria	Exclusion Criteria
ST-segment elevation myocardial infarction (ST elevation in two contiguous precordial leads and ischaemic chest pain).	Hemodynamically unstable patients (systolic blood pressure < 90 mmHg or mean arterial pressure < 60 mmHg).
Symptom onset within < 12 h.	Thrombolysis for the current event.
History of diabetes or glucose level at admission >180 mg/dL.	History of coronary artery bypass graft surgery.
Informed consent signed	Ongoing treatment with SGLT2 inhibitors.
Age 18–85 years	Known allergy to SGLT2 inhibitors.
Both sexes	History of recurrent urinary tract infections
	History of chronic kidney disease with an estimated glomerular filtration rate < 20 mL/min/1.73 m².
	For women of childbearing age: Current or planned pregnancy or lactation.
	Current Participation in another clinical trial or having participated in the week prior to recruitment

SGLT-2, sodium-glucose co-transporter 2.

### Study protocol

**Figure 1:**
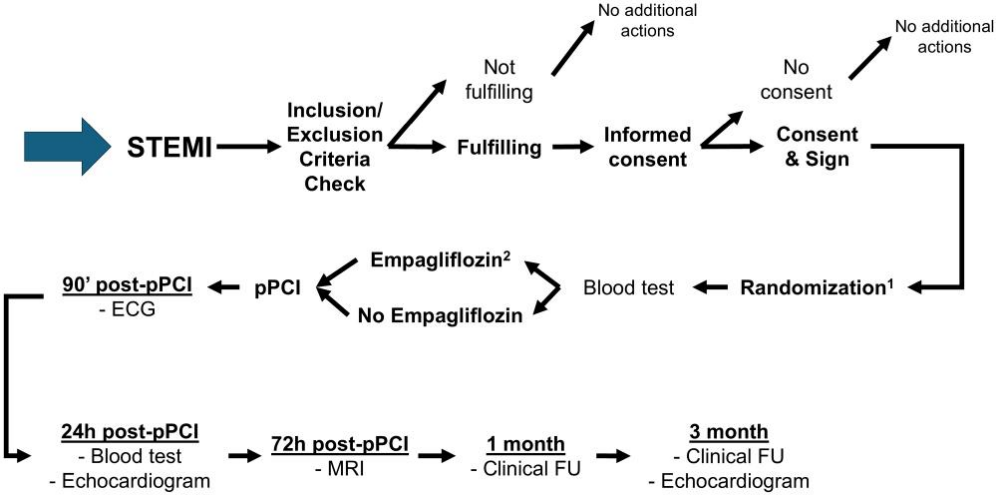
Flowchart of EMPA-PCI trial protocol. Stratified randomization will be used in order to ensure the balance between the two treatments in the strata defined by time from symptoms onset and infarct territory (4 strata). After the informed consent has been obtained form an eligible participant, investigators classify the subject into a particular stratum following a decision tree algorithm that gives an alpha-numeric classification code. The patient assigned to the empagliflozin group receives a loading dose of 25 mg orally, in addition to the standard STEMI treatment according to current guidelines. An electrocardiogram is performed 90 min after PCI, and blood tests are taken at 24, 48, and 72 h. An echocardiogram is performed at 24 h and at the 3-month follow-up. A magnetic resonance imaging scan is performed at 72 h, and clinical follow-up is conducted at 1 and 3 months.

The protocol flowchart is shown in *Figure 1*. Any patient who provides informed consent will be randomized to receive either empagliflozin plus standard treatment or standard treatment alone. The assigned intervention is provided at the earliest possible time point, and in all cases prior to primary percutaneous coronary intervention (PCI). The empagliflozin dosing regimen is specified in the legend of *Figure 1*. The study schedule is summarized in *Table 2*.

**Table 2:** Study schedule

Study Period									
	Enrolment	Allocation	Post-allocation						Close-out
Timepoint			Pre-PCI	PCI	Post-PCI (90 min)	Post-PCI (24 h)	Post-PCI (48 h)	Post-PCI (72 h)	Post-PCI (3 months)
**ENROLMENT:**									
Eligibility screen	X								
Informed consent	X								
Baseline characteristics	X								
Allocation		X							
**INTERVENTIONS:**									
Standard treatment			X			X	X	X	
Empagliflozin			X			X	X	X	
**ASSESSMENTS:**									
No-Reflow Phenomenon				X					
Blood samples			X			X	X	X	
Electrocardiography			X		X				
Echocardiography						X			X
Magnetic Resonance Imaging								X	
Adverse events				X		X	X	X	X
Information about study discontinuation									X

PCI, Percutaneous Coronary Intervention.

**Figure 2:**
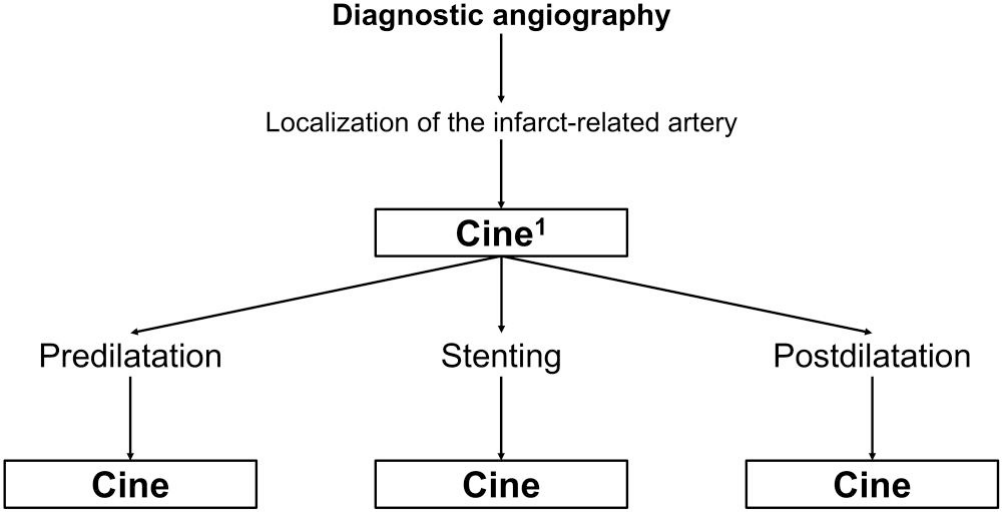
Angiography protocol in the Cath lab. The first angiography recorded for the study and that will be subjected to subsequent analysis will be the one in which the artery culprit of the infarction is located. In this recording the initial TIMI flow will be obtained. Subsequently, the angiography performed after each of the following moments will be recorded for protocol analysis: pre-dilation, stenting, post-dilation. Although these moments may or may not be present, depending on the operator's decision for each patient.

During primary PCI, standardized recordings will be performed at specific time points (*Figure 2*). Recordings may differ based on procedural characteristics and operator-dependent decisions. The presence of the no-reflow phenomenon will be assessed at each of these predefined time points. After the procedure, patients will be monitored in a coronary intensive care unit, where an electrocardiogram will be performed 2 h post-intervention. Beyond the protocol-defined treatment, additional medical management will be at the discretion of the attending cardiologist, who will also determine the appropriate timing for patient discharge. During hospitalization, laboratory tests will be performed at 24, 48, and 72 h post-procedure. A transthoracic echocardiogram with global longitudinal strain analysis will be conducted at 24 h, and a cardiac magnetic resonance imaging (MRI) scan will be performed up to 72 h after the intervention. During the study, the interventional cardiologists who perform the primary angioplasty, the doctors who perform the MRI, the doctors who perform the echocardiogram, those who follow-up the patients, the researchers who will perform the data analysis will be blinded to the treatment that the patients receive. Details regarding the study organization, MRI and echocardiography analysis, data collection, and the definition of clinical outcomes can be found in Supplementary material online, *Appendix A* and in the statistical analysis plan (SAP).

### Randomization

Patients will be individually assigned to either the empagliflozin treatment group or the control group receiving standard treatment. Randomization will be conducted using permuted block stratification based on four variables:

- Symptom duration (≤6 h; > 6 h).
- Infarct territory (anterior; non-anterior).

These variables were selected due to their independent impact on the no-reflow phenomenon and infarct size. For the randomization process, when a potential study candidate arrives at the emergency department, the investigator responsible for randomization will be contacted. This investigator will submit the relevant variables into a web-based randomization service.^14^ Once the treatment group is determined, the randomization investigator will communicate the assigned treatment to the site investigator, who will then administer the appropriate therapy.

### Angiographic analysis

During PCI, recordings will be performed at specific time points (*Figure 2*). These recordings may or may not be present in every angiographic study, as interventional treatment is at the operator’s discretion. The recordings will be send to an external core lab to determine the presence or absence of the no-reflow phenomenon. No-reflow will be assessed using the TIMI flow scale, and any flow grade other than TIMI 3 will be classified as no-reflow.

A transient no-reflow phenomenon will be defined as no-reflow occurring during the angioplasty procedure but resolving before its completion. In contrast, persistent no-reflow will be defined as a phenomenon persisting until the end of the procedure despite the administration of pharmacological interventions aimed at reversing it.

### Sample size calculation

The sample size calculation was based on the incidence of the no-reflow phenomenon, which averages 22.3%.^15^ Regarding the effect attributable to the intervention in reducing no-reflow, reductions of over 80% have been reported.^16^ However, for this study, we considered a more conservative reduction of 50%, leading to an expected incidence of 11.1% in the intervention group.

Using the GPower statistical software and Fisher’s exact test for hypothesis testing (two-tailed), we determined a sample size based on an 80% statistical power (1−*β*) and a significance level (*α*) of 0.05. This yielded an estimated sample size of 135 individuals. Considering a 20% increase to account for potential losses (*n* = 27), the final total sample size was set at 162 patients (81 per group). For further details on the sample size calculation, please refer to the supplementary SAP document.

### Statistical plan

This study is a randomized, parallel-group, observer-blinded clinical trial. For normally distributed data, summary statistics will be reported as means and standard deviations (SD). Log transformations will be applied to positively skewed data, and results will be back-transformed and reported as geometric means. If normal distribution is not achieved even after transformation, data will be reported as medians with interquartile ranges, and inferential analysis will be conducted using non-parametric or resampling methods (e.g. bootstrapping). Categorical data will be summarized as percentages.

All hypotheses will be tested using a two-sided significance level of 0.05. After data collection and completion of data management procedures (including data description, handling processes, transfer formats, and quality control), a preliminary blinded statistical analysis will be conducted to assess potential outliers, necessary data transformations, and the influence of baseline covariates not predefined in the SAP.

All data management and statistical analyses will be conducted using STATA 18 (or a more recent version). For further details on the statistical methodology, please refer to the supplementary SAP document.

### Statistical methods for primary outcome comparison

The primary analysis will follow an intent-to-treat approach, including all randomized STEMI patients with an available measurement for the primary outcome (angiographic no-reflow phenomenon). The main null hypothesis to be tested is that there is no difference between the two treatment arms in the incidence of the no-reflow phenomenon measured by angiography. The cumulative incidence of no-reflow will be compared between treatment groups using Fisher’s exact test. Subsequently, association measures such as relative risk and/or odds ratios (OR) will be estimated to quantify the effect of the intervention. A multivariable analysis will be performed using a mixed-effects logistic regression model to adjust for potential confounders in assessing the association.

We do not anticipate interactions between treatment effects and baseline covariates. Therefore, the study is designed to detect differences between the two treatment categories (main effects). However, as an exploratory analysis and in accordance with the ICH E9 guideline,^17^ additional linear models will be fitted to assess whether the efficacy of pre-PCI empagliflozin administration depends on baseline covariates such as ischaemia duration, sex, glucose, age, etc.

## Discussion

This study aims to determine whether administering empagliflozin prior to primary PCI provides an additional benefit beyond those already demonstrated in patients with AMI. Although large clinical trials have been conducted in this population, none have specifically explored the potential effect of empagliflozin on the no-reflow phenomenon or reperfusion injury.^18–20^ This is primarily because the main objective of those studies was to evaluate the impact on subsequent development of heart failure, and because the drug was not administered during the ischaemic period. (*Table 3*).

**Table 3:** Clinical trials of SGLT2 inhibitors in acute myocardial infarction

Trail	Inclusion criteria	Primary end point	Result	References
**The EMMY trial**	AMI and first intake of study medication **≤** **72 h AFTER AMI** after performance of a CA	Change in NT-ProBNP levels from randomization to week 26.	Positive	^18^
Empagliflozin 10 mg (*n* = 237) vs. placebo (*n* = 239)				
**DAPA-MI**	AMI within **10 days** before randomization AND Impaired LV systolic function or Q-wave MI.	Hierarchical composite of death, hospitalization for HF, non-fatal MI, AF/flutter event, new diagnosis of diabetes, NYHA FC at last visit and BW decrease of 5%	Positive	^19^
Dapagliflozin 10 mg (*n* = 2019) vs. placebo (*n* = 1988)				
**EMPACT-MI**	AMI within 14 days before randomization AND new LVEF <45% or congestion	Composite of hospitalization for HF or death from any cause.	Negative	^21^
Empagliflozin 10 mg (*n* = 3260) vs. placebo (*n* = 3262)				
	PLUS one additional enrichment factor			

AF, atrial fibrilation; AMI, acute myocardial infarction; BW, body weight; FC, functional class; HF, heart failure; LV, left ventricle; LVEF, left ventricle ejection fraction; MI, myocardial infarction.

In the past, several pharmacologic agents have been evaluated for their potential to mitigate reperfusion injury. Among them, adenosine,^22^ nitric oxide,^23^ sodium nitrate,^24^ and cyclosporine A^25^ failed to reduce infarct size in patients with AMI. This may be due to the fact that the mechanisms of action of these agents do not fully account for the complex pathophysiology of reperfusion injury.^26^ In contrast, other drugs such as metoprolol,^27^ the glucose–insulin–potassium (GIK) solution,^28^ exenatide,^29^ and abciximab^20^ have demonstrated a beneficial effect in preventing reperfusion injury. However, these therapies have not been widely adopted as standard treatment due to limitations in their practical use, restricted indications in STEMI patients, or because, despite reducing reperfusion injury, they failed to show significant clinical benefit.^30^

On the other hand, it is likely that empagliflozin exerts a beneficial effect on the no-reflow phenomenon and reperfusion injury. In recent years, several preclinical studies, not only with empagliflozin but also with other SGLT2 inhibitors, have shown that administration of these agents, even after the onset of ischaemia or during early reperfusion, significantly reduces these outcomes in ischaemia-reperfusion models.^30–34^ (*Table 4*).

**Table 4:** Preclinical evidence on SGLT2 inhibitors and reperfusion injury

Study	SLGT2 inhibitor	Timing of administration	Result	References
Andreadou I *et al*.^31^	Empa	6 weeks before ischaemia	↓ infarct size	^31^
Yu YW *et al*.^32^	Dapa	1 week before ischaemia	↓ infarct size	^32^
			↓ levels of cTnI, CK-MB and LDH	
Zou R *et al*.^33^	Empa	1 week before ischaemia	↓ levels of cTnI, CK-MB and LDH	^33^
Lahnwong C et al.^34^	Dapa	G1: 15 min before ischaemia **G2: 15 min into ischaemia**	G1: ↓ infarct size and ↓ arrhythmia score	^34^
			G2: ↓ infarct size	
		G3: Onset of reperfusion		
			G3: no difference	
Nikolaou PE *et al*.^13^	Empa	G1: 6 w before ischaemia	G1 y G2: ↓ infarct size, ↓ no-reflow, ↑ cardiac function	^13^
		**G2: Onset of reperfusion**		

CK-MB, Creatine Kinase MB; Dapa, Dapagliflozin; Empa, Empagliflozin; G1, group 1; G2, group 2; LDH, lactate dehydrogenase; w, weeks.

**Figure 3:**
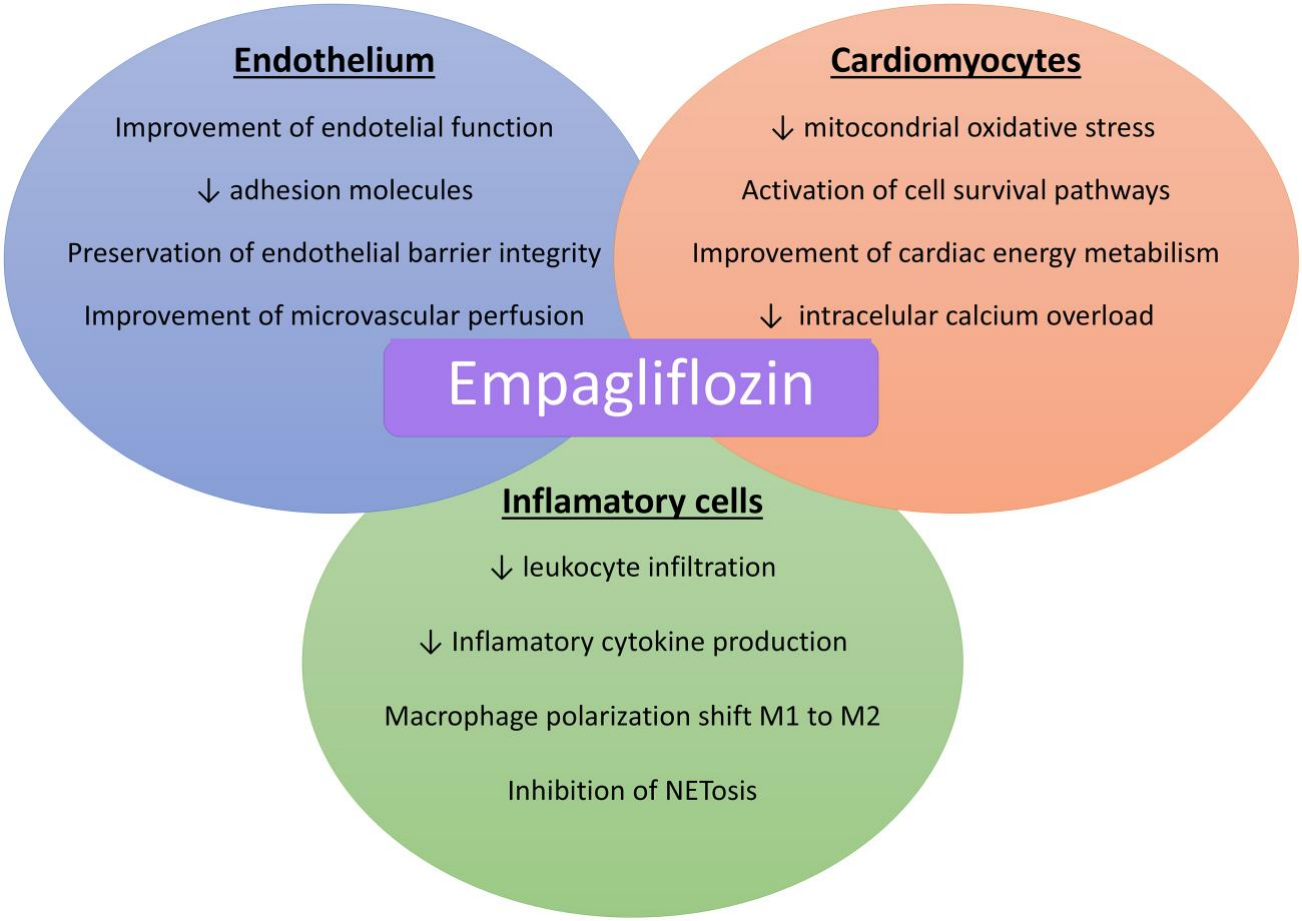
Mechanisms of action of empagliflozin in preventing reperfusion injury. Multiple preclinical trials using ischaemia-reperfusion models have demonstrated that empagliflozin reduces reperfusion injury by acting directly on cardiomyocytes, endothelial cells, and inflammatory cells.

One of the potential strengths of empagliflozin is that it does not act through a single mechanism of action; rather, it exerts effects at multiple levels involved in the pathophysiology of reperfusion injury. (*Figure 3*).

In cardiomyocytes, empagliflozin attenuates reperfusion injury through several mechanisms: reduction of reactive oxygen species via activation of the liver kinase B1 (LKB1)/AMPK signaling pathway, which decreases mitochondrial superoxide production;^35^ activation of pro-survival signaling pathways such as the Reperfusion Injury Salvage Kinase (RISK) pathway and the JAK/STAT pathway;^36^ improvement of energy metabolism through increased ketone body production, mediated by upregulation of proteins related to ketone metabolism, including monocarboxylate transporter 1 (MCT1), beta-hydroxybutyrate dehydrogenase 1, and succinyl-CoA:3-ketoacid CoA transferase;^37^ and reduction of calcium overload via regulation of Ca2+/calmodulin-dependent protein kinase II (CaMKII) expression and activity, which prevents calcium leakage from the sarcoplasmic reticulum into the cytoplasm.^38^

In the endothelium, empagliflozin contributes to the prevention of the no-reflow phenomenon and the reduction of reperfusion injury through several mechanisms: decreasing inflammatory cell recruitment by downregulating the overexpression of cell adhesion molecules such as intercellular adhesion molecule-1 (ICAM-1);^13^ improving endothelial function by preserving the balance between endothelin-1 (ET-1) and endothelial nitric oxide synthase; maintaining endothelial barrier integrity by preserving endothelial gap junctions;^33^ and enhancing microvascular perfusion by reducing *P*-selectin antigen expression on activated platelets and significantly decreasing P2Y12 reaction units (PRUs), molecules involved in microvascular thrombosis.^39^

In inflammatory cells, empagliflozin mitigates reperfusion injury through the following mechanisms: reduction of leukocyte infiltration via modulation of gene transcription involved in inflammatory molecule production;^13^ decreased production of cytokines such as IL-1, IL-6, and TNF-α, which is also associated with reduced leukocyte infiltration;^40, 41^ promotion of macrophage polarization from the pro-inflammatory M1 phenotype to the anti-inflammatory M2 phenotype, thereby contributing to inflammation resolution;^42^ and downregulation of high mobility group box 1 (HMGB1) expression, which significantly reduces the formation of neutrophil extracellular traps.^43^

One of the key strengths of the EMPA-PCI trial is that the effect of empagliflozin will be assessed through multiple modalities that have previously been used independently as surrogate markers of reperfusion injury, and have been associated with adverse clinical outcomes.^44^ Angiographic no-reflow, the trial’s primary endpoint, has been linked, both in its transient and persistent forms, to higher rates of reinfarction, increased bleeding, larger infarct size, impaired ventricular function, and ultimately, increased mortality.^45^

A key particularity of this study is the unconventional empagliflozin dosing regimen. This is justified by our aim to investigate the drug's immediate effect on reperfusion injury. While we are aware of existing evidence demonstrating the benefits of empagliflozin initiated days after myocardial infarction for long-term remodeling, our research specifically targets its earlier, cardioprotective effects. We hypothesize that this critical window extends up to the first 72 h after reperfusion, a pivotal period encompassing infarct expansion, necrosis, oedema, and neutrophil infiltration—processes directly linked to clinical outcomes.^46, 47^ Although evaluating these clinical outcomes is not the primary aim of the study, and the 3-month follow-up may not be optimal for this purpose, it is justified by the fact that most adverse events related to no-reflow and reperfusion injury occur within the first 30 days post-infarction.^48^

## Limitations

We acknowledge several limitations: The sample size may be underpowered for clinical outcomes; The 3-month follow-up is likely too short to capture major long-term events; The single-centre design limits generalizability; The open-label design (empagliflozin vs. standard care) may introduce bias, despite blinded endpoint adjudication; The use of adjunctive therapies (GPIIb/IIIa inhibitors, aspiration) was at operator discretion, potentially confounding results.

## Conclusion

Although the cardiovascular benefits of SGLT2 inhibitors are well established, it remains unknown whether their administration prior to primary PCI in STEMI patients can prevent the no-reflow phenomenon and attenuate reperfusion injury. This study has the potential to provide novel evidence regarding the use of empagliflozin in the setting of AMI and to demonstrate that its administration before revascularization may offer an additional clinical benefit in this patient population.

### Trial Status

Protocol version 3 (date); recruitment began on 17 August and is expected to be completed by mid-December.

## Lead author biography


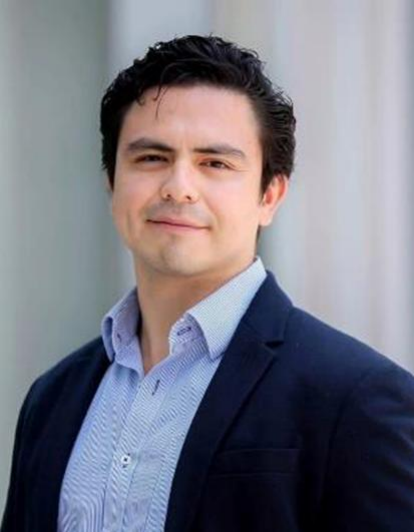


Dr Fabio Solis-Jimenez, MD, PhD(c), is a recently graduated interventional cardiologist from the National Institute of Cardiology Ignacio Chávez in Mexico City. He is currently undertaking a fellowship in structural interventional cardiology at Hospital Clínico San Carlos in Madrid and pursuing a PhD in Medical Sciences at the National Autonomous University of Mexico. He is considered an emerging researcher in the field of interventional cardiology. He received a grant from the Secretariat of Science, Humanities, Technology, and Innovation (Secihti) for the EMPA-PCI project, which constitutes his doctoral research and may provide the foundation for future studies on reperfusion injury.

## Supplementary Material

oeaf128_Supplementary_Data

## Data Availability

No new data were generated or analysed in support of this research.
