# Supplementary material for: Effect of empagliflozin on reducing the no-reflow phenomenon in patients with ST-elevation myocardial infarction: rationale and design of the EMPA-PCI trial
Source: Eur Heart J Open. 2025 Oct 5;5(6):oeaf128. doi: 10.1093/ehjopen/oeaf128 (PMC12604469; doi:10.1093/ehjopen/oeaf128)
Supplement: oeaf128_Supplementary_Data [file oeaf128_supplementary_data.zip › APPENDIX A.docx]

**APPENDIX A**

**Study Organization and Oversight**

The EMPA-PCI trial is overseen by a multi-tiered governance structure to ensure integrity, quality, and adherence to the protocol and statistical plan:

- Steering Committee: Meets regularly and oversees all strategic and scientific aspects of the trial.
- Executive Committee: A subset of the steering committee, responsible for the day-to-day operation and management of the trial.
- Independent Clinical Events Committee (CEC): Provided by the Instituto Nacional de Cardiología, this autonomous and independent committee is responsible for the blinded adjudication of all clinical endpoint events.
- Data and Safety Monitoring Board (DSMB): Constituted by the institution's Office for Systematic Support for Superior Research (OASIS), this independent board is responsible for the ongoing review of safety data and overall trial conduct.
- Imaging Committee: Supervises the acquisition quality, centralized analysis, and interpretation of all imaging studies (e.g., angiography, cardiac MRI, echocardiography), following pre-specified protocols detailed in the Statistical Analysis Plan (SAP).
- Statistical Team: Responsible for executing the pre-specified analyses outlined in the SAP, maintaining blinding, and ensuring the integrity of the data processing.

All researchers involved in the trial undergo regular accreditation courses in Good Clinical Practice (GCP) to ensure the protocol is executed to the highest standard. The study protocol and SAP have been reviewed and approved by all pertinent ethics and research committees and is registered on [ClinicalTrials.gov](https://clinicaltrials.gov/) (NCT06342141).

**Magnetic resonance imaging analysis**

MRI scans will be performed on a 1.5 Tesla Magnetom Sola scanner (Siemens, Germany) using a phased array cardiac coil. The scanning protocol is based on previously published guidelines. (1)

The protocol will include a functional study using ECG-triggered breath-hold segmented steady-state free precession (SSFP) cine images in long-axis and short views. Native T1 and T2 mapping sequences and post contrast T1 mapping will be acquired using Modified Look-Locker Imaging (MOLLI) sequences and motion correction (MOCO). To assess late gadolinium enhancement (LGE) and microvascular obstruction (MVO), breath-hold ECG-triggered 2D inversion-recovery (I-R) gradient echo sequences will be acquired between 10 and 15 min after the administration of gadolinium-based contrast medium [gadobutrol 0.1 mmol/kg of body weight (Gadovist, Bayer, Germany)] in the long- and short-axis projections according to standardized protocols.

All post processed functional parameters as well as inversion-recovery sequences will be post processed using CVI 42 software (Circle CVI, Canada). Manual tracing of the endocardial and epicardial contours in the short axis images will be performed. A region of interest (ROI) within the normal myocardium will be traced and automatically propagated to the other slices, with manual correction if necessary. The extent of LGE is defined as a threshold of +5 standard deviations (SD) from the signal intensity of remote normal myocardium. With the signal intensity of the normal myocardium as a reference, the software shows the size of the infarcted myocardium (mass and percentage of LV mass) and MVO. Myocardial LGE is expressed in grams (g) and as a percentage (%) of LV mass. MVO was defined as “hypoenhancement” within the infarcted area and was quantified by manual delineation, expressed in grams (g) and percentage (%) of LV mass. (2)

**Transthoracic Echocardiogram Image Analysis**

All subjects underwent a comprehensive 2D echocardiographic study. Studies were performed using a Vivid ultrasound machine (GE Vingmed Ultrasound AS, Horten, Norway) equipped with an M5S probe. All patients were examined in the left lateral decubitus position using grayscale second-harmonic 2D imaging, with adjustment of image contrast, frequency, depth, and sector size for adequate frame rate and optimal LV endocardial border visualization. Echocardiographic studies included three apical views (four-chamber, two-chamber, and long-axis) optimized for global longitudinal strain (GLS) analysis. For each view, three consecutive heart cycles were recorded with a frame rate ranging between 50 and 80 frames/sec. Data sets were stored digitally and analyzed offline.

Using STE, GLS was obtained by averaging the measures of each segment peak systolic longitudinal strain value. Timing of aortic valve closure was selected using continuos wave doppler of the aortic valve. Inclusion of LV segments for analysis required the approval by the operator and both position and width adjustment of the automatically drawn region of interest were performed when necessary. In patients with more than two inadequately tracked LV segments in one view, GLS and MD were no longer computed and they were excluded from analysis. Mechanical dispersion (MD) was defined as the standard deviation of the time from the peak of the R wave on the ECG to the peak negative strain using a 16 segment LV model. All calculations of MD and GLS were done using commercially available software package (Automated Function Imaging, GE Vingmed Ultrasound, Horten, NO).

**Definition of Clinical Outcomes**

- **Malignant Arrhythmia:** Documented ventricular fibrillation or sustained ventricular tachycardia (>30 seconds or requiring termination due to hemodynamic compromise).
- **Reinfarction:** Defined according to the Fourth Universal Definition of Myocardial Infarction. Requires the presence of ischemic symptoms and/or new ischemic ECG changes accompanied by a rise and/or fall of cardiac troponin values with at least one value above the 99th percentile upper reference limit. In the context of the early phase post-PCI, a significant increase (>20%) from a previous troponin level that is already elevated and stable or falling is required.
- **Rehospitalization:** Hospital admission for any cardiovascular cause following discharge from the index event.
- **Cardiogenic Shock:** A clinical condition characterized by persistent hypotension (systolic blood pressure <90 mmHg or mean arterial pressure <65 mmHg for >30 minutes) or the requirement of vasopressors/inotropes to maintain a systolic blood pressure ≥90 mmHg, **and** evidence of end-organ hypoperfusion (e.g., cold extremities, oliguria <30 mL/hr, or altered mental status).
- **Urgent Revascularization:** An unplanned repeat percutaneous or surgical revascularization procedure performed on the target or non-target vessel due to recurrent ischemia.

**Data Collection**

All patient recruitment will occur in the emergency department of the National Institute of Cardiology in Mexico City. All study-related procedures, including coronary angiography, laboratory testing, echocardiography, cardiac magnetic resonance imaging (MRI), and clinical follow-up, will be performed at the same institution to ensure consistency.

An independent researcher, who is not involved in patient recruitment, clinical care, or outcome assessment, will be responsible for the development and management of the central electronic database. This separation of duties ensures data integrity and minimizes potential biases during data entry and management.

1. Kramer CM, Barkhausen J, Bucciarelli-Ducci C, Flamm SD, Kim RJ, Nagel E. Standardized cardiovascular magnetic resonance imaging (CMR) protocols: 2020 update. J Cardiovasc Magn Reson. 2020 Feb 24;22(1):17.
2. Alkhalil M, Borlotti A, De Maria GL, Gaughran L, Langrish J, Lucking A, et al. Dynamic changes in injured myocardium, very early after acute myocardial infarction, quantified using T1 mapping cardiovascular magnetic resonance. J Cardiovasc Magn Reson. 2018;20(1):82.
