# Supplementary material for: Effect of empagliflozin on reducing the no-reflow phenomenon in patients with ST-elevation myocardial infarction: rationale and design of the EMPA-PCI trial
Source: Eur Heart J Open. 2025 Oct 5;5(6):oeaf128. doi: 10.1093/ehjopen/oeaf128 (PMC12604469; doi:10.1093/ehjopen/oeaf128)
Supplement: oeaf128_Supplementary_Data [file oeaf128_supplementary_data.zip › SAP.pdf]

Statistical Analysis Plan (SAP)

Effect of Empagliflozin on Reducing the No-Reflow Phenomenon in Patients with ST-Elevation Myocardial Infarction: Rationale and Design of the EMPA-PCI Trial

Principal Investigator	Fabio Solis-Jimenez MD MSc<br>PhD Candidate<br>Instituto Nacional de Cardiología Ignacio Chávez<br>Mexico City, Mexico

Protocol Identification Number	24 – 1423
ClinicalTrials.gov identifier	NCT0634214

Authors	Gabriela Melendez-Ramirez MD PhD and Fabio Solis Jiménez PhDc<br>Director of the Master's and Doctoral Program in Medical Sciences of the Instituto Nacional de Cardiologia Ignacio Chávez<br>Mexico City, Mexico

Version	2

1. Administrative Information

This Statistical Analysis Plan (SAP) describes the detailed statistical methodology for the analysis of the EMPA-PCI trial. This SAP is written to ensure that the analysis is conducted in accordance with the study protocol and good statistical practice, minimizing bias and ensuring reproducibility. This document is finalized before database lock and unblinding.

Any deviations from this plan will be described and justified in the final clinical study report.

2. Introduction and Study Objectives

2.1. Study Background

The EMPA-PCI trial is a randomized, parallel-group, observer-blinded clinical trial designed to evaluate the efficacy of a loading dose of empagliflozin (25 mg) administered prior to primary percutaneous coronary intervention (PCI), followed by a maintenance dose of 10 mg daily for the subsequent 3 days, compared to standard treatment alone in patients with ST-segment elevation myocardial infarction (STEMI). The rationale is based on the potential cardioprotective effects of SGLT2 inhibitors, which may mitigate ischemia-reperfusion injury and the no-reflow phenomenon.

2.2. Primary Objective

To test the hypothesis that the administration of empagliflozin prior to primary PCI reduces the incidence of the angiographic no-reflow phenomenon compared to standard treatment alone.

2.3. Secondary Objectives

To compare the effect of pre-PCI empagliflozin versus standard treatment on:

1. Infarct size assessed by cardiac magnetic resonance imaging (MRI) at 72 hours.
2. Myocardial salvage index, assessed by cardiac MRI (calculated as [Area at risk - Infarct size] / Area at risk).
3. The percentage of ST-segment resolution on electrocardiogram at 2 hours post-PCI.
4. Final epicardial coronary flow, assessed by the TIMI flow scale at the end of the PCI procedure.
5. Myocardial tissue-level perfusion, assessed by the Myocardial Blush Grade (MBG) at the end of the PCI procedure.
6. The peak and area under the curve (AUC) of creatine kinase (CK), CK-MB, and troponins.
7. The proportion of ventricular dysfunction, assessed by global longitudinal strain on echocardiography at 24 hours.
8. The composite clinical outcome of rehospitalization, malignant arrhythmias, cardiogenic shock, reinfarction, urgent revascularization, and all-cause mortality at 3 months.

2.4. Hypotheses

- **Primary Null Hypothesis (H0):** There is no difference in the incidence of the angiographic no-reflow phenomenon between the empagliflozin and standard treatment groups.
- **Primary Alternative Hypothesis (H1):** The incidence of the angiographic no-reflow phenomenon is lower in the empagliflozin group than in the standard treatment group.

3. Study Design Overview

3.1. Trial Design

A randomized, two-arm, parallel-group, observer-blinded clinical trial.

3.2. Interventions

- **Experimental Group:** A loading dose of empagliflozin 25 mg administered orally before primary PCI, followed by a maintenance dose of empagliflozin 10 mg daily for the subsequent 3 days, in addition to standard medical treatment.
- **Control Group:** Standard medical treatment alone before and after PCI, as per international guidelines.

3.3. Schedule of Assessments

Assessments will be conducted at baseline, during PCI, and at specified time points post-PCI (2h, 24h, 48h, 72h) and at 3 months, as detailed in the study protocol.

4. Overall Analysis Approach

The analysis will be conducted with a focus on the intention-to-treat principle. The primary endpoint will be analyzed using a logistic regression model. Secondary endpoints will be analyzed using appropriate statistical methods (e.g., linear regression, mixed models, survival analysis) as detailed in subsequent sections of this plan. All tests will be two-sided with a significance level of $\alpha=0.05$. The analysis will be performed using Stata version 18 or higher.

5. General Statistical Aspects

5.1. Overall Analytical Principle

The primary analysis for all efficacy endpoints will be performed on the Full Analysis Set (FAS), following the intention-to-treat (ITT) principle. Supportive analyses will be performed on the Per-Protocol Set (PPS). All safety analyses will be performed on the Safety Set (SS).

5.2. Significance Level and Multiplicity

The primary analysis will be tested at a two-sided significance level of $\alpha = 0.05$. No adjustment for multiplicity will be made for the primary endpoint. Adjustment for multiple comparisons for secondary endpoints will be considered using the Holm-Bonferroni

method to control the family-wise error rate, given the exploratory nature of these analyses. The hierarchy of testing for secondary endpoints will be pre-specified prior to database lock.

5.3. Randomization and Blinding

Randomization was performed using a web-based system with permuted blocks stratified by:

1. Symptom duration (≤6 hours vs. >6 hours)
2. Infarct territory (anterior vs. non-anterior)

The trial is observer-blinded. The interventional cardiologists, imaging analysts, outcome assessors, and statisticians will be blinded to treatment assignment until the final database is locked.

5.4. Handling of Missing Data

The pattern and extent of missing data will be thoroughly summarized before any primary analysis. The strategy for handling missing data is detailed below for each endpoint category. The results will be considered robust if the conclusions from the primary analysis and the corresponding sensitivity analyses are consistent.

- **5.4.1. Primary Endpoint: Angiographic No-Reflow (Binary)**
  - **Primary Analysis:** Complete-case analysis. Patients with missing angiographic data will be excluded.
  - **Sensitivity Analysis:** Multiple Imputation (MI) using chained equations (m=50) under the Missing at Random (MAR) assumption. The imputation model will include treatment group, stratification variables (symptom duration, infarct territory), and other prognostically important baseline covariates.
- **5.4.2. Secondary Endpoints: Single Measurement Continuous Variables**
  - *Infarct Size by MRI (72h), Global Longitudinal Strain by Echo (24h), Myocardial Salvage Index*
  - **Primary Analysis:** Complete-case Analysis of Covariance (ANCOVA), adjusting for baseline values if available (e.g., baseline systolic function) and stratification variables.
  - **Sensitivity Analysis:** Multiple Imputation (MI) for the missing outcome, followed by the same ANCOVA model on each imputed dataset, with results pooled.
- **5.4.3. Secondary Endpoints: Repeated Measures Continuous Variables**
  - *Peak and AUC of CK, CK-MB, Troponins (measured at 24h, 48h, 72h)*
  - **Primary Analysis:** Mixed-Effects Models for Repeated Measures (MMRM). The model will include fixed effects for treatment, time, treatment-by-time interaction, and stratification variables, with an appropriate covariance

structure to model within-patient correlations. This model uses all available data and provides valid inferences under the MAR assumption.

- **Sensitivity Analysis:** Not typically required for the primary analysis of these endpoints, as the MMRM is itself a principled method for handling missing data. The pattern of missingness will be investigated.

- **5.4.4. Secondary Endpoints: Binary Angiographic Outcomes**
  - *TIMI Flow Grade (dichotomized), Myocardial Blush Grade (MBG) (dichotomized)*
  - **Primary Analysis:** Complete-case analysis using logistic regression, adjusted for stratification variables.
  - **Sensitivity Analysis:** Multiple Imputation (MI) for the missing outcome, followed by the same logistic regression model on each imputed dataset.
- **5.4.5. Secondary Endpoint: Time-to-Event Composite Outcome**
  - *Composite of rehospitalization, arrhythmias, shock, reinfarction, revascularization, death (3 months)*
  - **Primary Analysis:** Kaplan-Meier estimator and Cox proportional hazards model. Patients lost to follow-up will be right-censored at the date of their last known contact.
  - **Sensitivity Analysis:** A sensitivity analysis will be conducted to assess the impact of competing risks (e.g., death) if necessary.

6. Analysis of Efficacy Endpoints

6.1. Primary Endpoint Analysis

- **6.1.1. Endpoint Definition:** The primary endpoint is the occurrence of the angiographic no-reflow phenomenon, defined as a TIMI flow grade ≤ 2 at the end of the PCI procedure, as assessed by an independent core lab.
- **6.1.2. Analytical Approach:** The analysis will follow the intention-to-treat (ITT) principle in the Full Analysis Set (FAS).
- **6.1.3. Statistical Model:**
  - **Primary Analysis (Unadjusted):** The proportion of patients with no-reflow will be compared between treatment groups using Fisher's exact test. The effect size will be expressed as an unadjusted Odds Ratio (OR) with a 95% confidence interval.
  - **Primary Analysis (Adjusted):** A multivariable logistic regression model will be used to estimate the treatment effect, adjusted for the stratification factors used in randomization: symptom duration (≤6 vs. >6 hours) and infarct territory (anterior vs. non-anterior). The results will be presented as an adjusted OR with a 95% CI and a p-value.

- **6.1.4. Handling of Missing Data:** Patients with missing primary endpoint data will be excluded from the primary analysis. A sensitivity analysis using multiple imputation will be performed to assess the impact of missing data (as detailed in Section 5.4.1).

6.2. Secondary Endpoints Analysis

The analysis of secondary endpoints will be performed on the FAS. No adjustment for multiplicity will be applied for hypothesis testing on secondary endpoints, as they are considered exploratory.

- **6.2.1. Infarct Size by Cardiac MRI (Continuous)**
  - **Analysis:** Analysis of Covariance (ANCOVA), adjusting for the stratification factors (symptom duration, infarct territory).
  - **Effect Measure:** Difference in least-squares means between groups with a 95% CI.
- **6.2.2. Myocardial Salvage Index by Cardiac MRI (Continuous)**
  - **Analysis:** Analysis of Covariance (ANCOVA), adjusting for the stratification factors.
  - **Effect Measure:** Difference in least-squares means between groups with a 95% CI.
- **6.2.3. ST-Segment Resolution (%) at 2 hours (Continuous)**
  - **Analysis:** Analysis of Covariance (ANCOVA), adjusting for the stratification factors.
  - **Effect Measure:** Difference in least-squares means between groups with a 95% CI.
- **6.2.4. TIMI Flow Grade (Ordinal/Binary)**
  - **Analysis:** The distribution of TIMI flow grades (0-3) will be compared using a Mann-Whitney U test (ordinal). Additionally, the proportion of patients with TIMI flow ≤ 2 will be compared using logistic regression, adjusted for stratification factors.
  - **Effect Measure:** Odds Ratio (OR) with 95% CI for the binary outcome.
- **6.2.5. Myocardial Blush Grade (MBG) (Ordinal/Binary)**
  - **Analysis:** The distribution of MBG grades (0-3) will be compared using a Mann-Whitney U test (ordinal). Additionally, the proportion of patients with MBG grade 0-1 will be compared using logistic regression, adjusted for stratification factors.
  - **Effect Measure:** Odds Ratio (OR) with 95% CI for the binary outcome.
- **6.2.6. Enzymatic Peak and AUC (Longitudinal)**

- **Analysis:** A **Mixed-Effects Model for Repeated Measures (MMRM)** will be used. The model will include fixed effects for treatment, time, the treatment-by-time interaction, and the stratification factors.
- **Effect Measure:** The model-derived estimated mean values at each time point for each treatment group, along with the difference between groups at specific time points (e.g., 72 hours) with 95% CIs.

- **6.2.7. Global Longitudinal Strain by Echo (Continuous)**
  - **Analysis:** Analysis of Covariance (ANCOVA), adjusting for the stratification factors.
  - **Effect Measure:** Difference in least-squares means between groups with a 95% CI.
- **6.2.8. Composite Clinical Outcome (Time-to-Event)**
  - **Analysis:** Time-to-first-event analysis will be performed using the Kaplan-Meier method and a Cox proportional hazards model. The proportionality assumption will be tested.
  - **Effect Measure:** Hazard Ratio (HR) with a 95% confidence interval and a log-rank test p-value.

6.3. Supportive and Sensitivity Analyses

- The primary endpoint will be re-analyzed in the Per-Protocol (PP)
- All supportive analyses detailed in Section 5.4 (Handling of Missing Data) will be conducted to assess the robustness of the findings for both primary and secondary endpoints.

7. Analysis of Safety Endpoints

7.1. Safety Analysis Set

All analyses of safety will be performed on the Safety Set (SS), defined as all randomized patients who received at least one dose of the study intervention (empagliflozin loading dose or standard treatment).

7.2. Overview of Safety Analysis

The analysis of safety will be primarily descriptive and exploratory. No formal hypothesis testing is planned for safety endpoints. The focus will be on estimating and reporting the incidence of adverse events, with differences between groups described using point estimates and confidence intervals to assess clinical relevance.

7.3. Definition of Adverse Events

- **Adverse Event (AE):** Any untoward medical occurrence in a patient administered a study intervention, which does not necessarily have a causal relationship with the treatment.

- **Serious Adverse Event (SAE):** Any AE that results in death, is life-threatening, requires inpatient hospitalization or prolongation of existing hospitalization, results in persistent or significant disability/incapacity, or is a congenital anomaly/birth defect.
- **Adverse Event of Special Interest (AESI):** Given the mechanism of action of empagliflozin, the following AESIs will be actively monitored and reported:
    - Genitourinary infections.
    - Volume depletion events (e.g., hypotension, dizziness).
    - Diabetic ketoacidosis (in patients with known diabetes or hyperglycemia).
    - Hypoglycemic events.

7.4. Analysis of Adverse Events

- **7.4.1. Incidence:** The number and percentage of patients experiencing at least one AE, SAE, and AESI will be summarized by treatment group.
- **7.4.2. Severity:** The maximum severity (e.g., mild, moderate, severe) of any AE experienced by a patient will be summarized using the Common Terminology Criteria for Adverse Events (CTCAE) scale, where applicable.
- **7.4.3. Relationship:** The investigator's assessment of the relationship to the study intervention (e.g., related, not related) will be summarized for all AEs.

7.5. Analysis of Laboratory Parameters

- **7.5.1. Summary:** Laboratory values (e.g., creatinine, eGFR, electrolytes, hematology) collected at predefined time points will be summarized using descriptive statistics (n, mean, SD, median, min, max) for each treatment group.
- **7.5.2. Shifts:** Shift tables from baseline to the worst post-baseline value will be presented to show clinically significant changes in laboratory parameters, using predefined reference ranges.

7.6. Analysis of Vital Signs and Other Assessments

Vital signs (e.g., blood pressure, heart rate) and other safety assessments (e.g., ECG findings) will be summarized descriptively by treatment group and time point. Clinically significant changes will be highlighted.

7.7. Presentation of Results

All safety data will be presented in summary tables and listings. The listings will include detailed information for all SAEs and AESIs. Differences in incidence between groups will be presented as risk differences with 95% confidence intervals to facilitate a clinical (rather than statistical) interpretation of any imbalances.

7.8. Safety and Endpoint Adjudication Oversight

7.8.1. Data Safety Monitoring Board (DSMB)

An independent monitoring committee, constituted by the Office for Systematic Support for Superior Research (OASIS) and serving as the Data Safety Monitoring Board (DSMB) for this trial, will provide safety oversight. The DSMB will receive unblinded reports of accumulated adverse events and efficacy data at pre-specified intervals. The statistical team will provide these reports, which will include summaries of events by treatment group, to the DSMB. The DSMB will operate under a dedicated charter defining stopping guidelines for safety or efficacy. All recommendations from the DSMB will be documented and provided to the Institutional Review Board (IRB).

7.8.2. Clinical Events Committee (CEC) and Blinding Oversight

As this is a clinical trial involving an investigational drug, the Instituto Nacional de Cardiología has provided an independent Clinical Events Committee (CEC), separate from the investigational team. The CEC is responsible for ensuring the unbiased adjudication of all clinical endpoints and for supervising the blinding strategies for all analyses.

- **Primary Function:** The CEC will perform centralized, blinded adjudication of all components of the composite clinical endpoint (rehospitalization, malignant arrhythmias, cardiogenic shock, reinfarction, urgent revascularization, and death) according to pre-specified definitions. The CEC members will be completely blinded to treatment allocation.
- **Blinding Supervision:** Furthermore, the CEC will oversee the implementation of blinding procedures for all core laboratory analyses, including but not limited to ECG (for ST-segment resolution) and CMR (for infarct size and myocardial salvage index). This ensures that all personnel responsible for these analyses are completely blinded to treatment allocation, patient identity, and clinical outcomes through rigorous data obfuscation and separation of duties from the clinical team.

8. Sample Size Consideration

8.1. Basis of Calculation

The sample size calculation was based on the primary endpoint of the study: the incidence of the angiographic no-reflow phenomenon.

8.2. Assumptions and Parameters

The following assumptions, derived from published literature, were used for the calculation:

- **Expected incidence in the Control Group (p_1):** 22.3%
- **Expected incidence in the Empagliflozin Group (p_2):** 11.1%
- **Statistical Power ($1 - \beta$):** 80%
- **Type I Error Rate (α):** 0.05 (two-sided)
- **Statistical Test:** Fisher's exact test

8.3. Calculation

Using the G*Power statistical software (version 3.1) with the parameters above, the initial calculated sample size required to detect this difference was N = 135 patients (approximately 67-68 per treatment group).

8.4. Adjustment for Attrition

To account for potential patient dropouts, protocol deviations, and missing primary endpoint data (e.g., unsuccessful angiographic recording), the sample size was increased by **20%**.

- Adjusted Sample Size: 135 x 1.20 = 162 patients
- This results in a target of N = 162 randomized patients, with 81 patients per treatment group.

8.5. Sensitivity Analisis of Statistical Power and Sample Size

Acknowledging that the assumed 50% relative risk reduction may be optimistic, a dual sensitivity analysis was conducted: 1) to evaluate the statistical power of the current sample size to detect more conservative effect sizes, and 2) to calculate the sample size that would have been required to power the study for those effects.

The table below outlines both scenarios, maintaining the control group incidence at 22.3% and the same statistical parameters (α=0.05, power=80%, two-sided Fisher's exact test).

Relative Risk Reduction	Incidencie in Empagliflozin Group	Absolute Risk Reduction	Statistical Power (N=162)	Sample Size Required for 80% Power
50%	11.1%	11.2%	80%	162
40%	13.4%	8.9%	65%	250
33%	15%	7.3%	51%	380
25%	16.7%	5.6%	35%	680

Table 1. Dual sensitivity analysis: Statistical power achieved with the final sample size (N=162) and the sample size required to achieve 80% power for detecting smaller effect sizes.

Interpretation and Implications:

- This analysis confirms that the study is adequately powered (80%) only for the base case effect size (50% RRR).
- The power diminishes rapidly for more conservative effect sizes (65% for 40% RRR, 51% for 33% RRR).
- To be powered at 80% for these more modest effects, the study would have required a significantly larger sample size (~250 for 40% RRR, ~380 for 33% RRR), which was not feasible within the scope of this trial.
- In other scenarios, a formal blinded sample size re-estimation could be considered to adjust recruitment goals based on observed interim parameters, while strictly preserving trial integrity and blinding.

- A negative result (non-significant p-value) in this trial must be interpreted with caution. It would not necessarily rule out a clinically meaningful benefit of empagliflozin of a smaller magnitude (e.g., a 33% or 40% RRR), as the study is underpowered to detect those effects. Conversely, a positive result would be a very strong finding, indicating a large treatment effect.

8.6. Final Enrollment Goal

The study will aim to enroll 162 patients to ensure that, even after accounting for an attrition rate of up to 20%, the analysis retains sufficient statistical power to test the primary hypothesis for the base case effect size.

9. Analysis of Eligibility and Disposition of Participants

9.1. Analysis Sets

The following analysis sets will be defined for the statistical analysis:

- **Screened Set:** All participants who provided informed consent for the study.
- **Full Analysis Set (FAS):** All randomized participants. The primary analysis for efficacy endpoints will be performed on the FAS following the intention-to-treat (ITT) principle.
- **Per-Protocol Set (PPS):** A subset of the FAS who completed the study without any major protocol deviations that could significantly affect the efficacy assessment. This set will be used for supportive analyses of the primary and secondary efficacy endpoints.
- **Safety Set (SS):** All randomized participants who received at least one dose of the study medication (empagliflozin or standard treatment). This set will be the primary population for all safety analyses.

9.2. Participant Flowchart

A participant flowchart, conforming to the CONSORT (Consolidated Standards of Reporting Trials) statement, will be generated to illustrate the flow of participants throughout each stage of the trial. This flowchart will include:

- The number of patients assessed for eligibility.
- The number of patients excluded, with specific reasons for exclusion (e.g., not meeting inclusion criteria, meeting exclusion criteria, declining to participate).
- The number of patients randomized to each treatment group.
- The number of patients who received the intended treatment.
- The number of patients who discontinued the study intervention and the primary reasons for discontinuation.
- The number of patients included in each analysis set (FAS, PPS, SS).

9.3. Eligibility Criteria Assessment

The assessment of eligibility will be performed during the screening period, prior to randomization. The complete list of inclusion and exclusion criteria is provided in Section 2.3.1 and Section 2.3.2 of this statistical analysis plan. A summary of the number and percentage of patients excluded for each major criterion will be presented.

9.4. Statistical Analysis of Baseline Characteristics

Baseline demographic and clinical characteristics will be summarized for the Full Analysis Set (FAS). Continuous variables will be presented using descriptive statistics (number of observations, mean, standard deviation, median, minimum, and maximum). Categorical variables will be summarized by frequencies and percentages. No formal statistical testing will be performed to compare baseline characteristics between treatment groups, as randomization is expected to balance these factors.

9.5. Protocol Deviations

All protocol deviations will be listed for each participant. The impact of major deviations on the efficacy results will be assessed by comparing the analyses from the FAS and the PPS. The frequency and type of protocol deviations will be summarized.
